# Improved Reproductive Health Equity Between the Poor and the Rich: An Analysis of Trends in 46 Low- and Middle-Income Countries

**DOI:** 10.9745/GHSP-D-15-00124

**Published:** 2015-09-07

**Authors:** John Ross

**Affiliations:** ^a^​ Demographic consultant.

## Abstract

While several indicators for reproductive health have improved for entire populations, few analyses are available for trends over time in the gaps between the poor and the rich. This paper tracks improvements in the equitable distribution of reproductive health indicators according to wealth quintiles, especially for contraceptive use, in 46 low- and middle-income countries based on national population-based surveys conducted between 1990 and 2013. It focuses on the gaps between the poorest and richest quintiles in the earliest and latest survey rounds across a number of reproductive health indicators related to family planning, fertility desires, antenatal care, and infant and child mortality, as well as on improvements in the absolute levels of contraceptive use by the poorest quintile. Gap changes were decomposed to show how the gaps can either diminish or grow due to either the bottom or top quintile, or both. In addition, bivariate correlation analysis was conducted to examine the relationship of the gaps, and of contraceptive use by the poor, to national family planning program efforts. Overall, the gaps between the poorest and richest have narrowed, due primarily to faster improvements among the poor than the rich. For example, the gap between the richest and poorest in the modern contraceptive prevalence rate has declined by 25%, from a 20.4 percentage point difference to a 15.4 point difference. And the gap has decreased more where family planning programs have been stronger. Across most of 18 other reproductive health indicators, the gaps have also been narrowing. For instance, the poor-rich gap for antenatal care decreased by over a third, from a difference of 30.7 percentage points to 19.6 percentage points. Gaps in infant and child mortality also have declined by about one-third. The pattern for contraceptive use in sub-Saharan Africa, however, has been mixed, with the gap actually increasing in some countries with strong programs. This disparity may largely reflect that family planning in the region is generally at an earlier stage in its history, and so programs may initially be reaching better-off clients, especially in urban areas. To promote additional equity, programs should emphasize efforts to increase access to voluntary family planning services to the least well-off, including those in rural and peri-urban areas.

## Abstract (precis)

In light of advocacy efforts to reach the poorest with better health services, an examination of recent history reveals that overall the poor-rich gap in contraceptive use is already narrowing substantially, and more so where family planning programs are stronger. For most of 18 other reproductive health indicators, the gap is also narrowing. However, contraceptive use gaps in many sub-Saharan African countries have not diminished, calling for strong family planning program efforts to improve equity.

## INTRODUCTION

While several indicators for reproductive health have improved for entire populations, the gaps between the poor and the rich are another matter. This article assesses the gaps between the poor and the rich regarding contraceptive use and selected reproductive health topics. The core question is whether the serious gaps between the poor and rich in measures of reproductive health have diminished over time and, if so, whether this is due to absolute improvements among the poor.

There is rather little literature on these topics. Some documentation is available on the actual differences among quintiles for reproductive health services. For example, Singh, Darroch, and Ashford show the systematic gradient across the 5 wealth quintiles for delivery in a health facility and for care for sexually transmitted infections (STIs) or STI symptoms.^1^ The series of national Demographic and Health Survey (DHS) reports include quintile differences for a range of indictors; see, for example, the initial tables on “Characteristics of Survey Respondents.”^2^ However, few analyses are available for trends over time in the poor-rich gaps.

Gakidou and Vayena, writing in 2007, compared modern contraceptive use by the poorest quintile with national averages of use.^3^ They found that modern contraceptive use by the poor remained quite low even while use nationally was rising: “Over time the gaps in use persist and are increasing.” The authors conclude, “The secular trend of increasing rates of modern contraceptive use has not resulted in a decrease of the gap in use …” While they did not study the trend or gaps for all contraceptive use, only modern use, at least as of 2007 the broad nature of the findings would probably not be much different for all use. However, the use of traditional methods may be relatively greater among the poor and among rural populations, which would lessen the gap for all use.

Another analysis, by Hosseinpoor and colleagues,^4^ argues that multiple indicators of health inequality should be used and that both gaps and absolute measures of inequality should be examined. Besides wealth quintiles, differences in residence, education, and gender should be considered to capture inequality. Moreover, since there are numerous dimensions to health inequality, proportional improvements in each should be the focus rather than any single standard. They illustrate the use of both absolute and relative inequalities in 30–31 countries for antenatal care received and births attended by health personnel, using DHS and other surveys conducted between 1993 and 2011 (9- to 11-year intervals between initial and latest surveys). That work is greatly expanded in a recent publication from the World Health Organization that includes interactive data visualization features, with an extensive analysis of some 23 indicators for reproductive health including maternal and child health.^5^ It covers 86 low- and middle-income countries, of which 42 have data on time trends. Four dimensions of inequality are traced, for economic status, education, residence, and gender, and countries are compared on a composite index of 8 of the 23 indicators. Contraceptive use is examined especially according to education differentials. In general, improvements over time have occurred faster among the least advantaged, but large gaps remain.

Improvements in reproductive health status over time have generally occurred faster among the least advantaged, but large gaps remain, finds a recent WHO study.

A predecessor of wealth quintiles is the possession of modern objects to assess personal socioeconomic status. This method was used as early as 1963, in a large-scale experiment of family planning adoption in Taichung, Taiwan. Freedman and Takeshita asked respondents whether they owned any of 9 objects such as a bicycle, radio, or sewing machine.^6^ The authors found close correlations between the number of modern objects owned and ever use of contraception or abortion, as well as attitudes toward traditional family values.

The purpose of this article, as stated above, is to examine wealth gaps over time in low- and middle-income countries regarding a number of reproductive health indicators, especially contraceptive use, using national population-based surveys conducted between 1990 and 2013.

## DATA AND METHODS

The data sets used here come from the DHS series, which offers standardized tabulations with wealth quintiles that are not available in other sources such as the Reproductive Health Surveys (RHS) or the Multiple Indicator Cluster Survey (MICS) series. All data were accessed online through the DHS Program STATcompiler (www.statcompiler.com), which contained 249 surveys as of April 2015. Of these, 62 lacked breakdowns by quintiles and 25 countries had only a single survey, leaving 162 surveys (for 46 different countries). The analyses here compare the earliest survey to the most recent survey in each country (total of 92 surveys) to capture the maximum time interval in which changes could be observed (Table 1).

**TABLE 1:** Countries and Survey Years Included in the Analysis

Region/Country	Initial Survey Year	Latest Survey Year	Region/Country	Initial Survey Year	Latest Survey Year
**sub-Saharan Africa**			**North Africa/West Asia**		
Benin	1996	2006	Armenia	2000	2010
Burkina Faso	1998-99	2010	Egypt	1995	2008
Cameroon	1991	2011	Jordan	1990	2009
Chad	1996-97	2004	Morocco	1992	2003-04
Côte d'Ivoire	1994	2011-12	**Central Asia**		
Eritrea	1995	2002	Kazakhstan	1995	1999
Ethiopia	2000	2011	**South & Southeast Asia**				
Gabon	2000	2012	Bangladesh	1993-94	2011
Ghana	1993	2008	Cambodia	2000	2010
Guinea	1999	2005	India	1992-93	2005-06
Kenya	1993	2008-09	Indonesia	1997	2012
Lesotho	2004	2009	Nepal	1996	2011
Madagascar	1997	2008	Pakistan	1990-91	2012-13
Malawi	1992	2010	Philippines	1993	2008
Mali	1995-96	2006	Viet Nam	1997	2002
Mozambique	1997	2011	**Latin America & Caribbean**				
Namibia	1992	2006-07	Bolivia	1994	2008
Niger	1998	2006	Colombia	1990	2010
Nigeria	1990	2008	Dominican Republic	1996	2007
Rwanda	1992	2010	Guatemala	1995	1998-99
Senegal	1997	2010-11	Haiti	1994-95	2012
Tanzania	1996	2010	Honduras	2005-06	2011-12
Uganda	1995	2011	Nicaragua	1998	2001
Zambia	1996	2007	Peru	1991-92	2012
Zimbabwe	1994	2010-11

The intervals between the paired surveys for each country varied, averaging 14 years of observation time. The set of 92 surveys occurred between 1990 and 2013, with 2002 as the midpoint. The interquartile range was between 11 years and 16.5 years, so half of the surveys were clustered within that interval of 5.5 years. The 46 countries included the largest ones of Bangladesh, India, Indonesia, Nigeria, and Pakistan as well as numerous middle-sized ones; altogether they contain two-thirds (67.3%) of the developing world’s population outside of China. (However, each country has equal importance in the averages below since the averages are not weighted by population size.)

Limitations of the data occur partly because STATcompiler offers quintile breakdowns only on selected variables; nevertheless the variety is fairly inclusive. Some country questionnaires omit questions on a variable so that a few tabulations are for only 42 to 45 countries rather than 46; for example, the question for “ever had sex” was omitted in several countries. A few measures of potential interest are not included for lack of available quintile breakdowns, for example, the proportion of demand for contraception not yet satisfied by modern methods.^7^

### Dependent Variables

The **contraceptive prevalence rate** (CPR), which includes use of any type of contraceptive method, was analyzed for currently married women ages 15–49. We also analyzed the **modern contraceptive prevalence rate** (mCPR), which includes all methods except the “traditional” ones of rhythm and withdrawal. Here, we follow the DHS specifications of modern methods; they are the sum of male and female sterilization, intrauterine devices (IUDs), oral contraceptive pills, injectables, implants, male and female condoms, diaphragm/foam/jelly, the lactational amenorrhea method (LAM), the Standard Days Method (SDM), and “other” modern methods. However, across all DHS surveys, 5 of these methods (pills, IUDs, injectables, male condoms, and female sterilization) account for 96% of all modern method use.

Poor-rich gaps were examined for the CPR, mCPR, and 18 other reproductive health indicators.

We also explored 18 additional indicators to document whether any progress has been made in past years across a broad range of reproductive health concerns. The 18 indicators, along with the DHS definitions, are:

1. **Antenatal care (ANC):** percent distribution of live births in the 3 years (or in some surveys, 5 years) preceding the survey by source of ANC during pregnancy; also percent distribution of women who had a live birth in the 3 (5) years preceding the survey by number of ANC visits and by the timing of the first visit. The analyses here use only the percentage who had no ANC visits.
2. **Signs of pregnancy complications:** among women receiving antenatal care, percentage informed of signs of pregnancy complications.
3. **Receipt of iron or syrup:** percentage of women with a live birth in the 3 (5) years preceding the survey who received iron tablets or syrup or anti-malarial drugs for the most recent birth.
4. **Receipt of tetanus injections:** percent distribution of last live birth in the last 3 (5) years preceding the survey by number of tetanus toxoid injections given to the mother during pregnancy.
5. **Place of delivery:** percent distribution of live births in the last 3 (5) years preceding the survey, by place of delivery.
6. **Assistance during delivery:** percent distribution of live births in the last 3 (5) years preceding the survey, by type of assistance during delivery.
7. **Distance problems:** percentage of women who reported they have big problems in accessing health care for themselves when they are sick, by type of problem.
8. **Perinatal mortality:** number of stillbirths and early neonatal deaths, for the 5-year period preceding the survey.
9. **Infant mortality**: percent of births dying within the first year of life.
10. **Under-5 mortality**: percent of births dying within the first 5 years of life.
11. **Total wanted fertility rate:** for the 3 years preceding the survey. Similar to the total fertility rate but excludes unwanted births.
12. **Total fertility rate:** for the 3 years preceding the survey.
13. **Want to limit childbearing:** percentage of currently married women who want no more children by number of living children.
14. **Ideal number of children:** percent distribution of currently married women by ideal number of children, according to number of living children.
15. **Ideal number of children at age 20–24**: ideal number of children for currently married women aged 20-24.
16. **Unmet need for family planning:** percentage of currently married women with unmet need for family planning, i.e., the percentage of women currently married or in union who are fecund and who desire to either stop or postpone childbearing, but who are not currently using a contraceptive method.
17. **Unmet need for limiting**: separates women with unmet need for those who wish to *stop* future childbearing.
18. **Unmet need for spacing**: separates women with unmet need for those who wish to *postpone* the next birth; usually defined as at least 2 years later.

### Independent Variables

One analysis in this article examines the relationship of gaps in contraceptive use to the **efforts of national family planning programs**. The latter are measured in a series of studies in some 80 developing countries through ratings by local experts and are available approximately 5 years apart from 1982 through 2014. Termed the Family Planning Program Effort Index, the ratings comprise 31 items under the 4 components of policies, services, evaluation, and access to methods. A total score is included as the average of the 31 items, as well as summary scores for each of the 4 components. Details are given in a series of publications on the various rounds.^8^

The Family Planning Program Effort Index measures program efforts across policies, services, evaluation, and access to methods.

The analysis here used the Family Planning Program Effort Index cycles from 1994 through 2009, and it focused on the total score as well as the specific access score as a more immediate measure of provision of contraceptive supplies and services to the general population.

In relating gaps in contraceptive use to program effort, it is awkward to align the dates for the 2 measures, since the program effort studies occurred at fixed dates whereas the gaps are measured in surveys that were conducted at different dates and at different intervals. The surveys occurred between 1990 and 2013, so one way to explore the relationships is to examine the correlations between the gaps and all of the program cycles from 1994 through 2009, which occurred at the approximate midpoints of many survey intervals. This was done in relation to both the CPR and to the mCPR.

The analysis of contraceptive use gaps and family planning program efforts considers the sub-Saharan African region separately from the other countries due to the region’s special character and because its levels of contraceptive use are low, which tends to constrain the size of gaps in use between the poor and the rich. Of the 46 countries, 25 were in sub-Saharan Africa and 21 elsewhere (8 in Latin America, 8 in South and Southeast Asia, 4 in North Africa/West Asia, and 1 in the Central Asia Republics). Those 21 countries outside sub-Saharan Africa were combined since the individual numbers were too small for separate estimates.

The **wealth index** is a composite measure of a household’s living standard, using its ownership of selected assets such as bicycles and televisions; materials used in the housing construction; and types of water access and sanitation facilities.^9^ The index itself is divided into quintiles, then each household is scored on each of the index’s components to obtain the overall rating. The overall rating assigns the household to 1 of the 5 parts of the index. (Because the quintiles are defined on the weighted index, the final country distribution can differ from 20% of households in each quintile; however, generally the differences are small.) Finally, the household rating is applied to each individual member. The various DHS reports compare the influence of wealth on various population, health, and nutrition indicators, as noted above. For example:

- In Nigeria, children from the wealthiest households are nearly 15 times more likely to be vaccinated than those from the poorest households (58% vs. 4% are fully immunized, respectively).^10^
- In the Philippines, 96% of women in the wealthiest households are assisted by a health professional at delivery, compared with only 42% of women in the poorest households.^11^
- In Tanzania, HIV prevalence is nearly 2 times higher among women in the wealthiest households (8.0%) compared with those in the poorest households (4.8%).^12^

The wealth index is particular to each country, and the 5 quintiles are relative to each other, not to any international standard. Since they are gauged within each country, the absolute levels of poverty in the poorest quintile can be different from those in a neighboring country. However, Winfrey and colleagues have produced a standardized version of the wealth index that allows for comparisons across time for countries and across countries for any point in time.^13^ (See Rutstein and Staveteig for an earlier analysis.^14^) Winfrey and colleagues have used the standardized version to disaggregate the contribution of improvements in family planning for women at particular levels of economic status versus the improvements in overall economic status. This unusual effort echoes the call by Hosseinpoor and colleagues^4^ for attention to cross-country criteria.

Unlike the intermediate quintiles, the top and bottom ones include the extreme cases. That is, the wealth range within the top group can contain exceptionally well-off persons, and the bottom group can contain the most desperately poor. All quintile comparisons in this article are between the poorest and the wealthiest quintiles; preliminary tabulations were done to broaden the view to compare the bottom 2 quintiles to the top 2 quintiles. Those results closely paralleled the ones for the bottom vs. the top; only the contrasts were reduced (softened) compared with those between the bottom alone and the top alone.

All 5 quintiles can be examined through other techniques that examine the interplay of all 5 quintiles, but this analysis uses the simpler approach of comparing the poorest and the richest, which also reflects the focus of current discussions. Any of the available measures of inequality can be studied in relationship to such determinants as the nation’s modernization or urbanization levels, the allocation of public resources, or other characteristics.

### Data Analysis

For each feature of reproductive health examined, the analysis started with the values for all 5 quintiles, in the initial and latest surveys for each country. Next, the differences between the bottom and top quintiles in the initial and latest survey rounds were calculated to highlight the gap between the poor and the rich, along with the improvement in the gap. Further analysis was then conducted to decompose the gap changes, to show how they can either diminish or grow, due to changes in either the bottom or top quintile. In addition, for contraceptive use, an analysis explored how large the absolute increase in use was among those in the poorest wealth quintile; for both the CPR and the mCPR, ratios of the starting level to the ending level were calculated for each country, and then mean and median ratios for each region and for all 46 countries combined were calculated. Bivariate correlation analysis was performed to explore the relationship between contraceptive use gaps and family planning program effort.

## FINDINGS

### Contraceptive Use by Wealth Quintile

**FIGURE 1:**
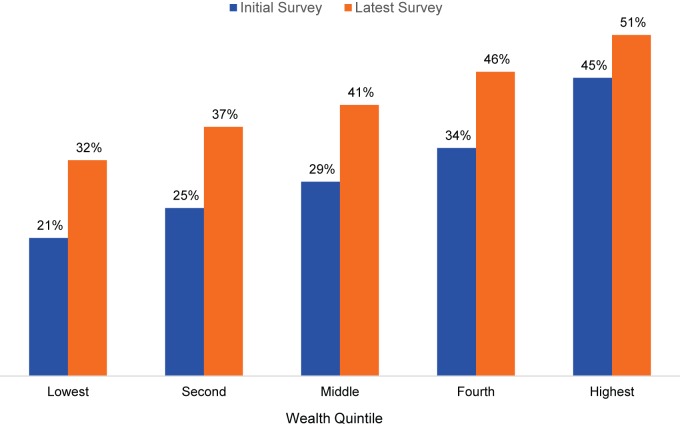
Contraceptive Prevalence Rates at Initial and Latest Surveys,^a^ by Wealth Quintile, 46 Low- and Middle-Income Countries ^a^ Survey years varied by country, ranging from 1990 in the initial surveys to 2013 in the latest surveys.

For all 46 countries included in the analysis, CPR increased with increasing wealth quintile (Figure 1). For example, among married women in the lowest quintile, the CPR was 21% in the initial survey rounds, and it grew to 32% in the latest surveys. Among the highest wealth quintile, the CPR was 45% in the initial surveys, growing to 51% in the latest surveys.

**FIGURE 2:**
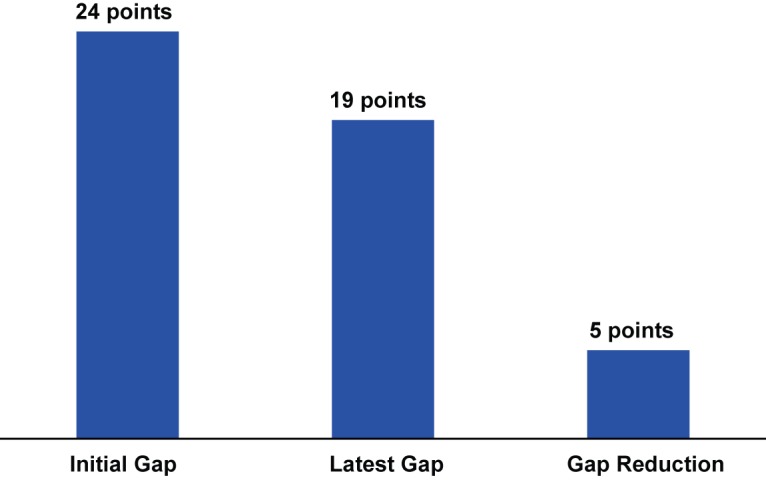
Gap (in Percentage Points) Between Richest and Poorest Quintiles in the Contraceptive Prevalence Rate, and Gap Reduction From Initial to Latest Survey,^a^ 46 Low- and Middle-Income Countries ^a^ Survey years varied by country, ranging from 1990 in the initial surveys to 2013 in the latest surveys.

The gap between the poor and the rich in contraceptive use has in fact declined over the years. In the initial survey rounds, the CPR gap between the highest and lowest wealth quintile was 24 percentage points (Figure 2). In the latest survey rounds, the CPR gap shrunk by 5 points, for a 19-percentage point difference between the highest and lowest wealth quintiles.

Gaps between the poor and the rich in contraceptive use have declined over the years.

### Decomposition of Gap Changes

In what ways can the gaps change, either growing or shrinking? A gap change is the net result of moves by either or both of the 2 quintiles being compared. It can come from an up or down move by the poor quintile and/or by the rich quintile.

Table 2 illustrates, for the CPR and mCPR, the ways in which a gap may increase (Ethiopia) or decline (Eritrea). The decomposition of the rising gap in Ethiopia shows CPR gains in both quintiles, but a far larger one in the rich quintile: 23.5 vs. only 9.8 in the poor quintile, so the gap increased by nearly 14 points. Eritrea illustrates a reverse dynamic, in which the CPR fell in both quintiles, but more so in the rich quintile: 5.1 vs. 0.8 in the poor quintile, shrinking the gap by 4.3 points. Parallel results occurred for the mCPR.

**TABLE 2:** Two Types of Changes^a^ in Quintile Gaps for Contraceptive Use, Illustrative Examples From Ethiopia and Eritrea

	Ethiopia: Rising Gaps			Eritrea: Declining Gaps		
	Gap at Initial Survey	Gap at Latest Survey	Gap Change	Gap at Initial Survey	Gap at Latest Survey	Gap Change
**CPR**						
Poor	3.5	13.3	9.8	2.5	1.7	−0.8
Rich	28.3	51.8	23.5	25.5	20.4	−5.1
Gap	24.8	38.5	13.7	23.0	18.7	−4.3
**mCPR**						
Poor	2.7	13.0	10.3	0.3	1.4	1.1
Rich	22.9	48.2	25.3	18.9	17.9	−1.0
Gap	20.2	35.2	15.0	18.6	16.5	−2.1

^a^​ The rising CPR and mCPR gaps in Ethiopia are a reflection of larger gains among the rich than among the poor. On the other hand, in Eritrea the CPR and mCPR fell among both quintiles but more so in the wealthiest quintile, resulting in shrinking gaps.

The type of change is decomposed for all 46 countries in Table 3. Countries are divided into those with increasing gaps (12 countries) and those with decreasing gaps (34 countries). The rows further separate countries by whether both rates fell, or both rose, or showed a mixed combination. In each cell, Q1 refers to the poorest quintile and Q5 to the richest quintile. For example, in the first row the CPR in both quintiles fell, but the CPR in the wealthiest quintile fell more, thereby shrinking the gap.

**TABLE 3:** List of Countries According to Type of Change in the CPR Gap and CPR Decrease or Increase Between Surveys, Q1 (Poorest) Compared With Q5 (Richest)

	Increasing CPR Gap	Decreasing CPR Gap		TOTAL
**Both rates fell**	**0**	**Q1 fell; Q5 fell more: 2**		**2**
		Eritrea		
		Senegal		
**Both rates rose**	**Q1 rose; Q5 rose more: 7**	**Q1 rose; Q5 rose less: 26**		**33**
	Ethiopia	Bangladesh	Kenya	
	Guatemala	Bolivia	Lesotho	
	Mozambique	Cambodia	Madagascar	
	Nigeria	Colombia	Malawi	
	Rwanda	Côte d'Ivoire	Morocco	
	Tanzania	Dominican Rep.	Namibia	
	Uganda	Egypt	Nepal	
		Guinea	Nicaragua	
		Haiti	Pakistan	
		Honduras	Peru	
		India	Philippines	
		Jordan	Zambia	
		Kazakhstan	Zimbabwe	
**Mixed**	**Q1 fell; Q5 rose: 5**	**Q1 rose; Q5 fell: 6**		**11**
	Armenia	Gabon		
	Benin	Ghana		
	Burkina Faso	Indonesia		
	Cameroon	Mali		
	Chad	Niger		
		Viet Nam		
**TOTAL**	**12**	**34**		**46**

The second row of Table 3 contains 33 countries, 7 cases in which the gap worsened as contraceptive use among the rich rose faster than among the poor. However, in the majority (26 of 46 countries, or 57%), the poor increased contraceptive use faster than the rich did. The third row shows 5 countries in which use fell among the poor and rose among the rich, enlarging the gap, together with 6 countries in which the opposite occurred, reducing the gap.

The poor increased contraceptive use faster than the rich did in the majority of countries examined.

In short, a diverse picture emerges for the ways in which the CPR gap can rise or fall over time. Mainly, however, the CPR gap has fallen as contraceptive use has increased faster among the poor than among the rich.

In absolute terms for the CPR itself, 39 of 46 cases (85%) can be viewed as having favorable results for the poor; the CPR rose among both poor and rich in 33 countries and among the poor but not the rich in 6 more.

Note that all countries with increasing gaps, in the first column, are in sub-Saharan Africa except Armenia. It is likely that contraceptive use has spread more rapidly in the cities than in the rural areas, which overlap with the bottom quintile. Again however, in absolute terms contraceptive use in 7 of the 12 countries has risen among both the poor and the rich.

### Gaps and Gap Improvements for Contraceptive Use

The gap between the richest and the poorest quintiles for contraceptive use has been shrinking overall, for both the CPR and the mCPR (Table 4, last row).

**TABLE 4:** Initial and Latest Gaps (in Percentage Points) Between Richest and Poorest Quintiles and Gap Changes Between Initial and Latest Survey, for the Contraceptive Prevalence Rate (CPR) and Modern Contraceptive Prevalence Rate (mCPR), 46 Low- and Middle-Income Countries

	CPR			mCPR		
Country, Latest Survey Year	Gap at Initial Survey	Gap at Latest Survey	Gap Change	Gap at Initial Survey	Gap at Latest Survey	Gap Change
**sub-Saharan Africa**						
Benin, 2006	17.0	25.9	−8.9	7.7	10.8	−3.1
Burkina Faso, 2010	16.7	30.0	−13.3	14.3	26.5	−12.2
Cameroon, 2011	31.2	38.3	−7.1	11.8	23.3	−11.5
Chad, 2004	6.6	10.3	−3.7	4.7	7.3	−2.6
Côte d'Ivoire, 2011–12	24.5	16.3	8.2	11.4	12.2	−0.8
Eritrea, 2002	23.0	18.7	4.3	18.6	16.5	2.1
Ethiopia, 2011	24.8	38.5	−13.7	20.2	35.2	−15.0
Gabon, 2012	23.1	14.9	8.2	12.6	10.0	2.6
Ghana, 2008	22.4	17.2	5.2	13.7	9.0	4.7
Guinea, 2005	13.4	11.8	1.6	8.2	10.0	−1.8
Kenya, 2008–09	37.0	34.6	2.4	34.8	31.0	3.8
Lesotho, 2009	36.9	31.6	5.3	37.8	32.3	5.5
Madagascar, 2008–09	39.9	37.4	2.5	21.5	18.8	2.7
Malawi, 2010	15.9	14.3	1.6	13.3	13.5	−0.2
Mali, 2006	19.2	15.4	3.8	14.8	13.6	1.2
Mozambique, 2011	16.8	27.4	−10.6	16.0	26.6	−10.6
Namibia, 2006–07	48.6	39.2	9.4	51.5	38.8	12.7
Niger, 2006	18.9	10.0	8.9	17.3	13.5	3.8
Nigeria, 2008	18.9	31.8	−12.9	11.7	19.8	−8.1
Rwanda, 2010	8.8	14.0	−5.2	6.3	11.1	−4.8
Senegal, 2010–11	20.6	19.7	0.9	22.6	18.5	4.1
Tanzania, 2010	26.3	27.7	−1.4	24.0	18.5	5.5
Uganda, 2011	22.3	31.5	−9.2	23.7	26.4	−2.7
Zambia, 2007	20.5	13.7	6.8	25.9	17.7	8.2
Zimbabwe, 2010–11	19.5	10.3	9.2	24.6	11.2	13.4
**Mean**	**22.9**	**23.2**	**−0.3**	**18.8**	**18.9**	**−0.1**
**North Africa/West Asia**						
Armenia, 2010	−2.4	9.2	−11.6	13.7	16.3	−2.6
Egypt, 2008	30.7	10.0	20.7	29.2	10.4	18.8
Jordan, 2009	30.0	11.8	18.2	22.8	12.6	10.2
Morocco, 2003–04	40.4	11.6	28.8	30.4	5.4	25.0
**Mean**	**24.7**	**10.7**	**14.0**	**24.0**	**11.2**	**12.9**
**Central Asia**						
Kazakhstan, 1999	12.8	4.5	8.3	5.8	6.2	−0.4
**South & Southeast Asia**						
Bangladesh, 2011	13.3	−0.7	14.0	8.2	−1.8	10.0
Cambodia, 2010	23.1	10.8	12.3	12.9	−3.8	16.7
India, 2005–06	29.7	25.3	4.4	25.7	23.4	2.3
Indonesia, 2012	13.7	5.1	8.6	10.7	2.4	8.3
Nepal, 2011	31.4	19.2	12.2	29.2	13.3	15.9
Pakistan, 2012–13	30.2	25.0	5.2	22.0	13.5	8.5
Philippines, 2008	17.4	9.2	8.2	15.7	7.2	8.5
Viet Nam, 2002	16.9	3.2	13.7	8.5	−6.3	14.8
**Mean**	**22.0**	**12.1**	**9.8**	**16.6**	**6.0**	**10.6**
**Latin America & Caribbean**						
Bolivia, 2008	45.7	24.6	21.1	39.9	23.9	16.0
Colombia, 2010	30.9	4.5	26.4	27.4	6.4	21.0
Dominican Rep., 2007	13.8	5.6	8.2	12.5	2.8	9.7
Guatemala, 1998–99	60.1	64.5	−4.4	51.7	54.3	−2.6
Haiti, 2012	23.6	1.3	22.3	16.0	−2.2	18.2
Honduras, 2011–12	20.0	8.8	11.2	23.5	12.3	11.2
Nicaragua, 2001	27.0	22.0	5.0	24.0	20.8	3.2
Peru, 2012	36.5	1.0	35.5	37.8	17.6	20.2
**Mean**	**32.2**	**16.5**	**15.7**	**29.1**	**17.0**	**12.1**
**Overall Mean**	**24.3**	**18.6**	**5.7**	**20.4**	**15.4**	**5.0**

For the CPR, the decline of 5.7 points against the starting level of 24.3 represents a 23% fall in the CPR gap, and the decline of 5.0 points on the starting mCPR level of 20.4 is a 25% fall in the mCPR gap. The average interval between surveys is 14 years, which converts the declines to 0.41 (5.7/14) and 0.36 points (5.0/14) per year, respectively.

The poor-rich gap for modern contraceptive use overall has declined by 25%.

Sub-Saharan Africa is different: the average gap change for its 25 countries has been nearly zero. There is diversity, however; 10 countries show a worsening gap for the CPR (12 for the mCPR), in some cases by a considerable margin of 10 or more points, while the other 15 countries show lessening gaps. The picture is mixed, as it is for the other regions.

The average gap change in contraceptive use overall in sub-Saharan Africa has been relatively static.

However, the other regions have experienced appreciable improvements in their gaps (leaving Central Asia aside with only Kazakhstan): changes of 14.0 (CPR) and 12.9 (mCPR) points in North Africa/West Asia, 9.8 and 10.6 points, respectively, in South and Southeast Asia, and 15.7 and 12.1 points, respectively, in Latin America and the Caribbean. Over 14 years, the annual paces for the CPR are 1.0 for North Africa/West Asia, 0.70 for South and Southeast Asia, and 1.1 for Latin America and the Caribbean, and 0.92, 0.76, and 0.86, respectively, for the mCPR (not shown).

Again, the picture is uneven: among countries, the smallest gap changes for the mCPR in these regions (excluding sub-Saharan Africa and Central Asia) range from -2.6 to 3.2; numerous others are about 8.0 to 11.2; and others exceed 15, with highs at 25.0 in Morocco, 21.0 in Colombia, and 20.2 in Peru.

Each measure, the CPR and the mCPR, has its own story to tell. Where the CPR gap closed more than the mCPR one did, equity for the use of traditional methods improved in addition to the improvement for modern methods, a welcome outcome. But where the mCPR gap improved more, the equity improvement for traditional methods was less; in fact if the CPR and mCPR gap reductions were equal there was no gap reduction in traditional method use. In Table 4, 13 of the 46 countries show a larger improvement for the mCPR than for the CPR, whereas in 16 other countries the CPR improvement was greater, reflecting an improvement for traditional methods in addition to that for modern methods. Most of the other countries show mixed patterns, with a worsening gap in either the CPR or mCPR or both. In some cases the CPR improved while the mCPR did not, so the improvement in the traditional method gap more than compensated for the worsening mCPR gap.

There is also substantial diversity in the sizes of the gaps themselves (not the gap changes), whether at the initial or latest surveys. Where contraceptive use has reached moderately high levels in the general population, the gap between rich and poor can be quite large, as in Kenya, Lesotho, Madagascar, and Namibia, ranging from 37 to nearly 50 points for the gaps in the CPR in the initial surveys, and none of these have shrunk very much.

Appendix 1 and Appendix 2 provide the full set of CPR and mCPR values, respectively, in the initial and latest surveys for each of the 46 countries by region, for the lowest and highest quintiles with the changes, the gap, and the gap reduction.

### Absolute Increases in Contraceptive Use by the Poorest Quintile

It is good news that the gaps in use between the poorest and wealthiest are narrowing, but how large is the absolute increase in use among the poorest? Gaps can narrow simply because contraceptive use declines among the wealthiest, as noted previously. Table 5 provides the absolute increases in use for both the CPR and the mCPR among those in the poorest quintile, as well as the ratios between the starting and ending levels, by region. Note that each ratio shown is the average of all 46 individual country ratios, so it does not necessarily reconcile with the overall starting and ending levels in each row. It is more accurate to rely upon the 46 ratios than upon the relation of the average starting level to the average ending level.

**TABLE 5:** Initial and Latest Levels of Contraceptive Use, by Region, for the Poorest Quintile

Region	Initial CPR Level	CPR Rise	Latest CPR Level	Ratio, Latest/Initial	Initial mCPR Level	mCPR Rise	Latest mCPR Level	Ratio, Latest/Initial^a^
Overall								
Mean	20.6	12.3	32.9	1.9	13.8	12.7	26.5	3.5
Median	14.6	10.6	31.8	1.7	5.5	10.3	24.3	2.6
sub-Saharan Africa								
Mean	9.5	7.6	17.1	2.0	4.6	9.6	14.2	4.2
Median	8.0	4.5	13.3	2.0	2.1	6.3	11.6	3.9
North Africa/West Asia								
Mean	34.0	21.1	55.1	2.0	19.2	21.2	40.3	2.1
Median	27.4	27.1	54.5	2.0	16.7	22.6	44.0	2.1
Central Asia								
Kazakhstan	52.8	10.9	63.7	1.2	43.8	5.1	48.9	1.1
South & Southeast Asia								
Mean	31.7	16.0	47.7	1.7	25.7	13.5	39.2	1.8
Median	28.3	13.2	43.7	1.5	20.3	10.6	35.4	1.6
Latin America & Caribbean								
Mean	33.6	19.1	52.8	1.9	24.1	18.2	42.3	3.8
Median	38.9	18.8	53.7	1.8	24.1	18.2	45.1	2.4

Abbreviations: CPR, contraceptive prevalence rate; mCPR, modern contraceptive prevalence rate.

^a^​ These ratios average the 46 individual country ratios. They do not reconcile with the average starting and ending levels in the same row since those smooth out the overall numerators and denominators of the ratios. It is preferable to use the 46 individual country ratios.

Clearly, the poorest quintile has substantially increased its contraceptive use across regions. Focusing just on the mCPR:

- On average, the mCPR has risen by 13 points, from 14% of married women using a modern method to 27%. (Total use has risen from 21% to 33%.)
- Overall, the average ratio of the ending-use level to the starting-use level is 3.5 (mean) or 2.6 (median). Note that these are averages across the individual ratios for the 46 countries, which include some quite high ratios, so the median is preferred (see footnote in Table 5 concerning ratios).
- Modern methods account for nearly all of the increase in contraceptive use. The average rise in modern use, of 12.7 points, slightly exceeds the average rise in all use of 12.3 points. That means that, on average, any substitution of modern for traditional use has been tiny, only 0.4 point.
- Sub-Saharan Africa is special since its starting-use levels were so far below those of the other regions: only 4.6% of married women used a modern method (mean) or 2.1% (median). It increased those levels about fourfold between starting and ending. However, a large proportionate increase is easier from a low starting level since a small absolute rise can become a large proportion. Most other regions started much higher and their absolute increases were much larger, yet their ratios are well below those of sub-Saharan Africa, except for the mean of 3.8 in Latin America.
- For total use (CPRs), the ratios are generally lower, principally because the starting levels are higher than for modern use (and in sub-Saharan Africa, the increases are less than for modern methods).
- Individual countries show substantial variation around each summary (Appendix 1).

More use of modern methods accounts for nearly all the increase in contraceptive use across regions.

### Family Planning Program Effort and Gaps in Contraceptive Use

Can the efforts of national family planning programs reduce gaps in contraceptive use, perhaps by helping the poor increase use faster than among the rich? Bivariate correlation analysis was performed to explore this relationship (Table 6).

**TABLE 6:** Correlations (*r* Values) for Relation of Family Planning Program Efforts to Gap Changes Between Richest and Poorest Quintiles and to Contraceptive Use by the Poorest, 46 Low- and Middle-Income Countries

	Gap Change			
	CPR	mCPR	CPR for Poorest Quintile	mCPR for Poorest Quintile
**sub-Saharan Africa (N=25 Countries)**				
Total Effort Score				
2009	0.58	0.49	0.43	0.43
2004	(0.04)	0.12	0.13	0.21
1999	0.26	0.39	0.36	0.42
1994	0.38	0.45	0.43	0.44
** Mean**	**0.30**	**0.36**	**0.34**	**0.38**
Access Score				
2009	0.55	0.52	0.60	0.61
2004	0.09	0.25	0.24	0.29
1999	0.34	0.49	0.38	0.41
1994	0.19	0.39	0.42	0.43
** Mean**	**0.29**	**0.41**	**0.41**	**0.44**
**All Other Countries (N=21 Countries)**				
Total Effort Score				
2009	0.25	0.41	0.32	0.47
2004	0.19	0.29	0.29	0.50
1999	0.20	0.22	0.28	0.36
1994	(0.02)	—	0.28	0.38
** Mean**	**0.16**	**0.23**	**0.29**	**0.43**
Access				
2009	0.32	0.35	0.44	0.53
2004	0.05	0.01	0.45	0.56
1999	0.32	0.09	0.40	0.26
1994	(0.11)	(0.25)	0.22	0.23
** Mean**	**0.15**	**0.05**	**0.38**	**0.40**

A positive “r” correlation value indicates that a greater gap reduction accompanies greater program effort, generally due to a faster increase in use by the poorest then the richest quintile, shrinking the disparity between the groups.

In **sub-Saharan Africa**, several points stand out (top of Table 6):

- In the top panel, most correlations are of substantial size, suggesting greater gap reductions accompanying greater program effort. The gaps have narrowed more where program effort has been stronger.
- Most correlations are closer with the mCPRs than with the CPRs, which reflects the focus of national programs on only modern methods.
- Most mCPR correlations are greater with the “Access Scores” than with the “Total Effort Scores” in both 2004 and 2009; they are about equal in 1994 and 1999. Normally we would expect correlations between access and contraceptive use to be relatively close since access is a necessary condition for contraceptive use and the two are intimately connected. However, correlations to gaps rather than levels are a different matter.
- The correlations with the 2004 scores are small, which is something of an anomaly since the correlations before, in 1999, and after, in 2009, are sizeable.
- To augment the analysis, we can examine actual use levels by the poorest quintile in relation to program effort; this follows on the evidence presented previously that the absolute levels of contraceptive use by the poor have risen. Since most gap reductions are due to a faster rise in contraceptive use among the poor than among the rich, how does use by the poor relate to program effort? The right panel of the sub-Saharan Africa section of Table 6 shows the positive relationship, with substantial correlations in all years, although less so for effort in 2004. Where program effort is stronger, use by the poorest quintile is higher. That in turn has translated into the shrinking gaps in contraceptive use between the poor and the rich, as mentioned previously.

Greater family planning program effort was correlated with greater gap reductions in contraceptive use.

For **all other countries** (lower part of Table 6), the patterns are generally similar, although with less regularity. We place less emphasis on these correlations due to small numbers in each subregion; however, the correlations for the relation of gap reductions to program effort are mostly positive, as they are for access. For unclear reasons, the figures for 1994 are weak, and even negative for access. Greater regularity and higher correlations appear in the right panel of the “all other countries” section, for more contraceptive use by the poor where programs are stronger. The pattern is consistent across all years.

Overall, the starting hypothesis is supported in both regions, that stronger program effort is accompanied by larger reductions in the gaps and by more contraceptive use among the poor that helps explain the gap reductions.

Finally, it is important to recognize that the poorest quintile is dominated, in most countries, by the rural population. The changes noted previously appear to occur disproportionately: “rural” and “poorest quintile” overlap considerably, as seen from two perspectives: (1) the percentage of the total rural population that falls into the bottom quintile, and (2) the percentage of the bottom quintile that is composed of rural people.

To illustrate, with examples taken from 3 DHS surveys in different regions, from the first perspective, in Indonesia, Egypt, and Malawi, 33%, 30%, and 23% of the rural population, respectively, fall into the poorest quintile. The second perspective is more telling: of all women in the poorest quintile, a remarkable 84%, 94%, and 98% live in rural areas, respectively. These high percentages reflect the features of which the wealth index is composed. Rural people are generally disadvantaged when it comes to the indicators of which the wealth index is composed. Appendix 3 provides fuller details. When we say “bottom quintile,” we are most likely saying “rural.” As contraceptive use, for example, has risen in the bottom quintile, it has risen primarily among rural residents.

### Gaps for Additional Indicators of Reproductive Health

The previous sections have focused in detail on contraceptive use; now we look at 18 additional reproductive health indicators for patterns in the changing gaps between the bottom and top quintiles.

Among the 18 indicators, most gaps have shrunk, while a few have not changed and 2 have increased (Table 7). In nearly all cases, the initial gap occurs because the poor rating was higher than the rich rating (so nearly all figures are positive). The exception is with desire to limit childbearing, since the rich typically want to limit further childbearing more than the poor do, and the rich are slightly more likely than the poor to say that the last birth was wanted later or not at all.

**TABLE 7:** Gaps Between the Poorest and Richest Quintiles (Poor Minus Rich) at Initial and Latest Survey^a^ and Gap Changes, by Reproductive Health Indicator, 46 Low- and Middle-Income Countries

Reproductive Health Indicator	Gap at Initial Survey	Gap at Latest Survey	Gap Reduction	% Improvement
% with no antenatal care	30.7	19.6	11.1	36.1
% not told of pregnancy complications	27.9	20.9	7.0	25.1
% not receiving iron or syrup	31.7	24.3	7.4	23.2
% not receiving tetanus	21.9	14.8	7.1	32.3
% not delivering in health facility	48.3	49.1	(0.7)	(1.6)
% lacking skilled attendant at birth	51.4	46.8	4.6	8.9
% with distance problem	52.6	35.5	17.1	32.5
Perinatal mortality	14.4	9.6	4.8	33.1
Infant mortality rate	37.1	24.2	13.0	34.9
Under-5 mortality rate	66.8	46.8	20.0	30.0
Total fertility rate	2.9	2.9	0.03	0.9
Wanted fertility rate	2.1	1.9	0.2	8.8
Want to limit childbearing	(7.1)	(2.5)	4.6	64.4
Ideal no. of children	1.38	1.28	0.10	7.4
Ideal no. of children at ages 20–24	1.17	1.02	0.14	12.0
Unmet need for family planning	7.9	7.8	0.1	1.4
Unmet need for limiting	3.7	3.6	0.2	5.1
Unmet need for spacing	4.1	4.2	(0.1)	(1.4)

^a^​ Survey years varied by country, ranging from 1990 in the initial surveys to 2013 in the latest surveys.

Note: Data in the last 2 columns were calculated with more decimals than shown and can reflect rounding.

The overriding finding is that the gaps have diminished on most indicators, which is good news for the field of reproductive health, although the annual pace of improvement may have been slow. In Table 7, the final column shows the percentage declines in the gaps. The annual pace of improvement is not shown; on average, for the 46 countries the interval between surveys is 14 years, but depending on the particular country the interval can be less or more, affecting the annual rate of change in the gap.

For lack of antenatal care, in the initial survey, the percentage of poor women who lacked ANC altogether was 30.7 percentage points higher than for rich women (pertains to births within the last 3 years). By the time of the most recent surveys, that gap had declined to 19.6 percentage points. Thus, the gap declined by over one-third (11.1/30.7).

Gaps in lack of antenatal care have declined by over one-third.

The gains have been positive and substantial for the next 3 pregnancy-related services, for being told of possible pregnancy complications, receiving iron or syrup (or in some countries also malaria tablets), and receiving tetanus shots. Next, an exception is delivering in a health facility, but interestingly, the gap for having a skilled attendant at birth has improved, perhaps reflecting more services at home among the poor. Distance as a serious barrier during sickness has improved sharply, again by about a third (17.1/52.6). Those gains are echoed in narrowed infant and child mortality gaps; they too have declined by about a third.

The gaps between the poor and the rich for the total fertility rate have remained unchanged, at 2.9 births, but the gap in the wanted fertility rate has declined slightly. The original rates that compose the gaps have fallen considerably (not shown); as the earlier sections explain, a gap can increase, decrease, or remain constant while the constituent rates behind the gaps move in various directions.

In Table 7, the gap can be negative if the rich rating is above the poor rating, as with “wanting to limit childbearing” (7.1%), since more of the rich wish to limit than the poor do. Then if later the gap narrows, the change itself is positive. Also, where the gap is small to begin with the absolute change is necessarily small, but the percentage change can be large, as also with the item for wanting to limit childbearing. The gaps are small for the ideal number of children and have not varied much over the years, either for all women or for young women aged 25–29. The gap for “ever had sex” is consistent with an earlier onset of sexual activity among the poor.

Finally, gaps in unmet need for family planning have changed very little, while in many countries unmet need overall has declined. All the unmet need gaps are subject to differing rates of change by the poor and the rich, which in turn can reflect a moving balance between increased contraceptive use and a decline in number of children wanted.

## DISCUSSION

Data from 46 low- and middle-income countries show equity improvements across a surprisingly wide range of reproductive health features. Differences have been shrinking between the poor and the rich for contraceptive use, for 5 of 6 major indicators of antenatal and delivery care, for reduced distances to urgent health services, and for infant/child mortality. The gaps have declined as well for the wanted fertility rate and, in smaller degrees, for ideal number of children and unmet need for limiting births. Methodologically, it is straightforward for any country to update or retrace its quintile gaps from a new survey or a review of past ones, and given the data, provincial comparisons are possible.

Equity between the poor and the rich has improved across a wide range of reproductive health indicators.

Gap improvements are not to be confused with general improvements for populations at large. The share held by the poor can remain constant or worsen as overall population rates improve. However, the findings here are that the declining gaps found in many indicators are due to faster relative increases by the poor than by the rich. That reflects improvements in the absolute levels for the poor, which can be separate and even more significant than the gap reductions.

Declining gaps across many indicators are due to faster relative increases by the poor than by the rich.

Overall the gaps in the indicators examined have diminished, but the diversity is considerable among regions and, within each region, among countries. For a few indicators, in some countries the gaps have worsened. Especially for the poorest quintile, some sub-Saharan African countries have not done well. However, even in such cases there may be internal contrasts in the levels (rather than the gaps) that suggest some progress, particularly if the levels have risen in each sector. For example, in Nigeria the CPR in cities was about 3 times higher than in the rural sector (25.9% vs. 9.4%, respectively) in 2008, and both had risen from the 2003 survey (16.7% urban and 6.5% rural, also a threefold difference).

### Programmatic Implications

The analyses above clarify how gap changes can occur in a mechanical sense, i.e., by whether the relative shift is faster in one quintile than another, with changes in either or both quintiles, to go up or down. Behind those trends, however, stand the actual reasons for the substantive changes. Where any quintiles, but especially the poor ones, show improvements, what are the reasons? For contraceptive use, the evidence is that national family planning programs are helpful, and they tend to focus on the general population rather than just on the upper wealth quintiles. It is heartening that the “equity gap” in contraceptive use is narrowing in most countries and narrowing more where program efforts, especially for access to methods, are stronger. This calls for continued and increasing program efforts to advance contraceptive use and to provide more equitable services. However, sub-Saharan Africa is a partial exception, with the overall gap remaining rather constant. And in some countries with notably strong family planning programs (e.g., Ethiopia and Rwanda), the gap actually increased over this time period. This disparity, however, may largely reflect that family planning in sub-Saharan Africa is in general at an earlier stage in its history, in which there is typically more demand for contraception among those who are better off. Further, early program efforts tend to focus on those easiest to reach, particularly in urban areas. And as we have seen, a lack of wealth is highly correlated with rural residence, and sub-Saharan Africa is more highly rural than any other region. As programs mature, they are able to serve a broader proportion of the population.

Thus, these findings reinforce the need for strong voluntary family planning programming to reduce gaps further. Where programs are deliberately focused on the rural or the poor, that reinforces the tendency for the bottom to improve faster than the top. The “equity gap” remains sizable in many countries, and programs should emphasize efforts to provide access to the least well-off, including those in rural areas and the poorer parts of urban areas. Examples include mobile outreach and social franchising approaches, which are especially successful in reaching rural and poorer clients,^15^^,^^16^ community health workers, vouchers, and use of the “total market approach,” which seeks to encourage the better-off to use private-sector services so as to free up public-sector services for less well-off clients. Meanwhile, the poor have been doing better over the past period of about 14 years, although the pace of doing so has been uneven across indicators. Overall, however, the narrowing poor-rich gaps across multiple indicators is rather remarkable.

Strong family planning programming is needed to reduce gaps between the poor and rich further.

**APPENDIX 1:** Contraceptive Prevalence Rate (CPR), CPR Changes, Quintile Gaps, and Changes in Quintile Gaps Between Initial and Latest Survey, 46 Low- and Middle-Income Countries

	Lowest Quintile		Highest Quintile			
Country, Survey Year	CPR	CPR Rise	CPR	CPR Rise	Quintile Gap	Gap Change
**sub-Saharan Africa**
Benin 2006	7.7	−1.5	33.6	7.4	25.9	−8.9
Benin 1996	9.2		26.2		17.0	
Burkina Faso 2010	7.4	−0.6	37.4	12.7	30.0	−23.6
Burkina Faso 1998–99	8.0		24.7		16.7	
Cameroon 2011	2.9	−1.9	41.2	5.2	38.3	−7.1
Cameroon 1991	4.8		36.0		31.2	
Chad 2004	0.0	−3.5	10.3	0.2	10.3	−3.7
Chad 1996–97	3.5		10.1		6.6	
Côte d'Ivoire 2011–12	11.7	8.0	28.0	−0.2	16.3	8.2
Côte d'Ivoire 1994	3.7		28.2		24.5	
Eritrea 2002	1.7	−0.8	20.4	−5.1	18.7	4.3
Eritrea 1995	2.5		25.5		23.0	
Ethiopia 2011	13.3	9.8	51.8	23.5	38.5	−13.7
Ethiopia 2000	3.5		28.3		24.8	
Gabon 2012	21.3	2.5	36.2	−5.7	14.9	8.2
Gabon 2000	18.8		41.9		23.1	
Ghana 2008	14.2	2.4	31.4	−2.8	17.2	5.2
Ghana 1993	11.8		34.2		22.4	
Guinea 2005	5.3	3.6	17.1	2.0	11.8	1.6
Guinea 1999	1.7		15.1		13.4	
Kenya 2008–09	20.1	5.4	54.7	3.0	34.6	2.4
Kenya 1993	14.7		51.7		37.0	
Lesotho 2009	30.1	12.5	61.7	7.2	31.6	5.3
Lesotho 2004	17.6		54.5		36.9	
Madagascar 2008–09	19.9	14.1	57.3	11.6	37.4	2.5
Madagascar 1997	5.8		45.7		39.9	
Malawi 2010	38.7	29.3	53.0	27.7	14.3	1.6
Malawi 1992	9.4		25.3		15.9	
Mali 2006	3.7	2.4	19.1	−1.4	15.4	3.8
Mali 1995–96	1.3		20.5		19.2	
Mozambique 2011	2.9	1.6	30.3	12.2	27.4	−10.6
Mozambique 1997	1.3		18.1		16.8	
Namibia 2006–07	31.9	22.4	71.1	13.0	39.2	9.4
Namibia 1992	9.5		58.1		48.6	
Niger 2006	10.9	7.3	20.9	−1.6	10.0	8.9
Niger 1998	3.6		22.5		18.9	
Nigeria 2008	3.2	2.3	35.0	15.2	31.8	−12.9
Nigeria 1990	0.9		19.8		18.9	
Rwanda 2010	43.1	23.6	57.1	28.8	14.0	−5.2
Rwanda 1992	19.5		28.3		8.8	
Senegal 2010–11	4.8	−1.8	24.5	−2.7	19.7	0.9
Senegal 1997	6.6		27.2		20.6	
Tanzania 2010	22.9	12.8	50.6	14.2	27.7	−1.4
Tanzania 1996	10.1		36.4		26.3	
Uganda 2011	14.7	4.5	46.2	13.7	31.5	−9.2
Uganda 1995	10.2		32.5		22.3	
Zambia 2007	40.5	20.5	54.2	13.7	13.7	6.8
Zambia 1996	20.0		40.5		20.5	
Zimbabwe 2010–11	54.3	14.2	64.6	5.0	10.3	9.2
Zimbabwe 1994	40.1		59.6		19.5	
**MEAN CHANGES**		**7.6**		**7.9**		**−0.3**
**North Africa/West Asia**
Armenia 2010	53.2	−8.4	62.4	3.2	9.2	−11.6
Armenia 2000	61.6		59.2		−2.4	
Egypt 2008	55.4	25.0	65.4	4.3	10.0	20.7
Egypt 1995	30.4		61.1		30.7	
Jordan 2009	53.5	29.1	65.3	10.9	11.8	18.2
Jordan 1990	24.4		54.4		30.0	
Morocco 2003–04	58.3	38.6	69.9	9.8	11.6	28.8
Morocco 1992	19.7		60.1		40.4	
**MEAN CHANGES**		**21.1**		**7.1**		**14.0**
**Central Asia**
Kazakhstan 1999	63.7	10.9	68.2	2.6	4.5	8.3
Kazakhstan 1995	52.8		65.6		12.8	
**South & Southeast Asia**
Bangladesh 2011	61.5	21.1	60.8	7.1	−0.7	14.0
Bangladesh 1993–94	40.4		53.7		13.3	
Cambodia 2010	45.2	30.8	56.0	18.5	10.8	12.3
Cambodia 2000	14.4		37.5		23.1	
India 2005–06	42.2	14.1	67.5	9.7	25.3	4.4
India 1992–93	28.1		57.8		29.7	
Indonesia 2012	56.2	7.9	61.3	−0.7	5.1	8.6
Indonesia 1997	48.3		62.0		13.7	
Nepal 2011	40.4	22.7	59.6	10.5	19.2	12.2
Nepal 1996	17.7		49.1		31.4	
Pakistan 2012–13	20.8	19.2	45.8	14.0	25.0	5.2
Pakistan 1990–91	1.6		31.8		30.2	
Philippines 2008	40.8	12.3	50.0	4.1	9.2	8.2
Philippines 1993	28.5		45.9		17.4	
Viet Nam 2002	74.3	10.2	77.5	−3.5	3.2	13.7
Viet Nam 1997	64.1		81.0		16.9	
**MEAN CHANGES**		**17.3**		**7.5**		**9.8**
**Latin America & Caribbean**
Bolivia 2008	46.2	23.6	70.8	2.5	24.6	21.1
Bolivia 1994	22.6		68.3		45.7	
Colombia 2010	75.5	29.0	80.0	2.6	4.5	26.4
Colombia 1990	46.5		77.4		30.9	
Dominican Rep. 2007	67.5	12.7	73.1	4.5	5.6	8.2
Dominican Rep. 1996	54.8		68.6		13.8	
Guatemala 1998–99	8.4	1.8	72.9	6.2	64.5	−4.4
Guatemala 1995	6.6		66.7		60.1	
Haiti 2012	31.7	24.1	33.0	1.8	1.3	22.3
Haiti 1994–95	7.6		31.2		23.6	
Honduras 2011–12	67.3	14.0	76.1	2.8	8.8	11.2
Honduras 2005–06	53.3		73.3		20.0	
Nicaragua 2001	52.5	10.3	74.5	5.3	22.0	5.0
Nicaragua 1998	42.2		69.2		27.0	
Peru 2012	72.9	37.4	73.9	1.9	1.0	35.5
Peru 1991–92	35.5		72.0		36.5	
**MEAN CHANGES**		**19.1**		**3.5**		**15.7**
**OVERALL MEAN CHANGES**		**12.5**		**6.8**		**5.4**

**APPENDIX 2:** Modern Contraceptive Prevalence Rate (mCPR), mCPR Changes, Quintile Gaps, and Changes in Quintile Gaps Between Initial and Latest Survey, 46 Low- and Middle-Income Countries

	Lowest Quintile		Highest Quintile			
Country, Survey Year	mCPR	mCPR Rise	mCPR	mCPR Rise	Gap	Gap Change
**sub-Saharan Africa**
Benin 2006	2.4	1.1	13.2	4.2	10.8	−3.1
Benin 1996	1.3		9.0		7.7	
Burkina Faso 2010	7.1	6.3	33.6	17.2	26.5	−10.9
Burkina Faso 1998–99	0.8		16.4		15.6	
Cameroon 2011	2.4	1.7	25.7	13.2	23.3	−11.5
Cameroon 1991	0.7		12.5		11.8	
Chad 2004	0.0	−0.1	7.3	2.5	7.3	−2.6
Chad 1996–97	0.1		4.8		4.7	
Côte d'Ivoire 2011–12	7.4	6.3	19.6	7.1	12.2	−0.8
Côte d'Ivoire 1994	1.1		12.5		11.4	
Eritrea 2002	1.4	1.1	17.9	−1.0	16.5	2.1
Eritrea 1995	0.3		18.9		18.6	
Ethiopia 2011	13.0	10.3	48.2	25.3	35.2	−15.0
Ethiopia 2000	2.7		22.9		20.2	
Gabon 2012	11.9	6.3	21.9	3.7	10.0	2.6
Gabon 2000	5.6		18.2		12.6	
Ghana 2008	11.6	6.2	20.6	1.5	9.0	4.7
Ghana 1993	5.4		19.1		13.7	
Guinea 2005	2.7	1.7	12.7	3.5	10.0	−1.8
Guinea 1999	1.0		9.2		8.2	
Kenya 2008–09	16.9	6.6	47.9	2.8	31.0	3.8
Kenya 1993	10.3		45.1		34.8	
Lesotho 2009	28.6	13.2	60.9	7.7	32.3	5.5
Lesotho 2004	15.4		53.2		37.8	
Madagascar 2008–09	17.6	15.3	36.4	12.6	18.8	2.7
Madagascar 1997	2.3		23.8		21.5	
Malawi 2010	34.9	31.0	48.4	31.2	13.5	−0.2
Malawi 1992	3.9		17.2		13.3	
Mali 2006	2.8	2.3	16.4	1.1	13.6	1.2
Mali 1995–96	0.5		15.3		14.8	
Mozambique 2011	2.9	2.0	29.5	12.6	26.6	−10.6
Mozambique 1997	0.9		16.9		16.0	
Namibia 2006–07	29.6	24.2	68.4	11.5	38.8	12.7
Namibia 1992	5.4		56.9		51.5	
Niger 2006	2.3	1.5	15.8	−2.3	13.5	3.8
Niger 1998	0.8		18.1		17.3	
Nigeria 2008	2.5	2.0	22.3	10.1	19.8	−8.1
Nigeria 1990	0.5		12.2		11.7	
Rwanda 2010	38.5	26.7	49.6	31.5	11.1	−4.8
Rwanda 1992	11.8		18.1		6.3	
Senegal 2010–11	4.4	3.4	22.9	−0.7	18.5	4.1
Senegal 1997	1.0		23.6		22.6	
Tanzania 2010	19.2	14.3	37.7	8.8	18.5	5.5
Tanzania 1996	4.9		28.9		24.0	
Uganda 2011	12.7	10.6	39.1	13.3	26.4	−2.7
Uganda 1995	2.1		25.8		23.7	
Zambia 2007	30.6	25.2	48.3	17.0	17.7	8.2
Zambia 1996	5.4		31.3		25.9	
Zimbabwe 2010–11	52.4	21.2	63.6	7.8	11.2	13.4
Zimbabwe 1994	31.2		55.8		24.6	
**MEAN CHANGES**		**9.6**		**9.7**		**−0.1**
**North Africa/West Asia**
Armenia 2010	21.4	5.9	37.7	8.5	16.3	−2.6
Armenia 2000	15.5		29.2		13.7	
Egypt 2008	51.9	23.7	62.3	4.9	10.4	18.8
Egypt 1995	28.2		57.4		29.2	
Jordan 2009	36.6	21.5	49.2	11.3	12.6	10.2
Jordan 1990	15.1		37.9		22.8	
Morocco 2003–04	51.4	33.5	56.8	8.5	5.4	25.0
Morocco 1992	17.9		48.3		30.4	
**MEAN CHANGES**		**21.2**		**8.3**		**12.9**
**Central Asia**
Kazakhstan 1999	48.9	5.1	55.1	5.5	6.2	−0.4
Kazakhstan 1995	43.8		49.6		5.8	
**South & Southeast Asia**
Bangladesh 2011	52.9	18.4	51.1	8.4	−1.8	10.0
Bangladesh 1993–94	34.5		42.7		8.2	
Cambodia 2010	35.2	22.7	31.4	6.0	−3.8	16.7
Cambodia 2000	12.5		25.4		12.9	
India 2005–06	34.6	9.7	58.0	7.4	23.4	2.3
India 1992–93	24.9		50.6		25.7	
Indonesia 2012	53.0	6.8	55.4	−1.5	2.4	8.3
Indonesia 1997	46.2		56.9		10.7	
Nepal 2011	35.6	19.9	48.9	4.0	13.3	15.9
Nepal 1996	15.7		44.9		29.2	
Pakistan 2012–13	18.1	16.9	31.6	8.4	13.5	8.5
Pakistan 1990–91	1.2		23.2		22.0	
Philippines 2008	25.9	10.2	33.1	1.7	7.2	8.5
Philippines 1993	15.7		31.4		15.7	
Viet Nam 2002	57.9	10.9	51.6	−3.9	−6.3	14.8
Viet Nam 1997	47.0		55.5		8.5	
**MEAN CHANGES**		**14.4**		**3.8**		**10.6**
**Latin America & Caribbean**
Bolivia 2008	22.6	21.0	46.5	5.0	23.9	16.0
Bolivia 1994	1.6		41.5		39.9	
Colombia 2010	68.5	30.8	74.9	9.8	6.4	21.0
Colombia 1990	37.7		65.1		27.4	
Dominican Rep. 2007	66.5	15.3	69.3	5.6	2.8	9.7
Dominican Rep. 1996	51.2		63.7		12.5	
Guatemala 1998–99	5.4	0.0	59.7	2.6	54.3	−2.6
Guatemala 1995	5.4		57.1		51.7	
Haiti 2012	29.7	24.8	27.5	6.6	−2.2	18.2
Haiti 1994–95	4.9		20.9		16.0	
Honduras 2011–12	55.1	14.0	67.4	2.8	12.3	11.2
Honduras 2005–06	41.1		64.6		23.5	
Nicaragua 2001	50.2	10.0	71.0	6.8	20.8	3.2
Nicaragua 1998	40.2		64.2		24.0	
Peru 2012	40.5	30.0	58.1	9.8	17.6	20.2
Peru 1991–92	10.5		48.3		37.8	
**MEAN CHANGES**		**18.2**		**6.1**		**12.1**
**OVERALL MEAN CHANGES**		**12.9**		**7.8**		**5.0**

**APPENDIX 3:** Distribution of Survey Respondents by Quintile and Urban-Rural Residence

	Quintile					
Country, Survey Year	1	2	3	4	5	
**Indonesia, 2012**						**Total**
Urban	6.4	13.6	21.0	26.4	32.7	100
Rural	33.5	26.4	19.0	13.7	7.4	100
						
Urban	16.0	34.0	52.5	65.8	81.5	
Rural	84.0	66.0	47.5	34.2	18.5	
**Total**	**100.0**	**100.0**	**100.0**	**100.0**	**100.0**	
**Egypt, 2014**						**Total**
Urban	3.1	2.8	4.6	35.8	53.7	100
Rural	30.0	30.2	29.2	10.6	0.0	100
						
Urban	5.6	5.1	8.3	66.1	100.0	
Rural	94.4	94.9	91.7	33.9	0.0	
Total	100.0	100.0	100.0	100.0	100.0	
**Malawi, 2010**						**Total**
Urban	2.9	3.4	7.5	19.9	66.3	100
Rural	23.3	23.1	22.3	20.0	11.3	100
						
Urban	2.3	2.7	5.9	15.7	52.4	
Rural	97.7	97.3	94.1	84.3	47.6	
**Total**	**100.0**	**100.0**	**100.0**	**100.0**	**100.0**	

Note: The first “Urban” and “Rural” rows under each country represent the percentage of the urban and rural population, respectively, falling under each wealth quintile. The second set of “Urban” and “Rural” rows under each country represent the percentage of respondents from each wealth quintile who live in urban or rural areas, respectively.

**APPENDIX FIGURE 3:**
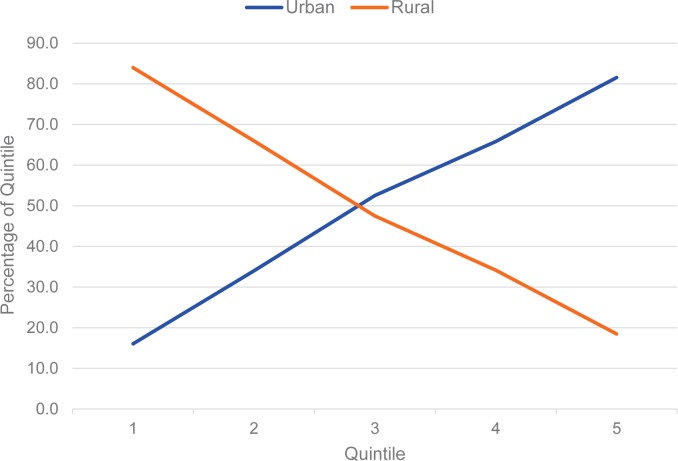
Quintile Membership by Rural and Urban Residence: Illustrative Example From Indonesia, 2012

## Notes

**Peer Reviewed**
